# Fatty infiltration of the gluteus medius and minimus muscles: volumetric analysis of both hips in patients with unilateral greater trochanteric pain syndrome using 2-point-Dixon MRI

**DOI:** 10.1186/s13244-025-02175-3

**Published:** 2025-12-22

**Authors:** Georg Wilhelm Kajdi, Sophia Samira Goller, Patrick Oliver Zingg, Reto Sutter

**Affiliations:** 1 Department of Radiology, Balgrist University Hospital, Faculty of Medicine, University of Zurich, Zurich, Switzerland; 2 Department of Orthopedics, Balgrist University Hospital, Faculty of Medicine, University of Zurich, Zurich, Switzerland

**Keywords:** Greater trochanteric pain syndrome, Gluteus medius, Gluteus minimus, Fat fraction, Dixon MRI

## Abstract

**Objectives:**

To investigate normal and pathologic values of fatty infiltration (FI) and muscle volume through volumetric quantification of the main hip abductors of patients with unilateral greater trochanteric pain syndrome (GTPS) using 2-point-Dixon MRI.

**Materials and methods:**

Patients prospectively underwent MRI of both hips: FI of the gluteus minimus (Gmin) and medius (Gmed) muscles were quantified by volumetric fat fractions (3D FF) using 2-point-Dixon MRI. Whole (WMV) and lean muscle volumes (LMV) were calculated for both muscles. 3D FF and volumes were compared between asymptomatic and GTPS hips, using the Wilcoxon signed-rank test. Gender-specific differences were assessed using the Mann–Whitney U test.

**Results:**

Forty-one patients (mean age 65.0 ± 13.6 years, 27 females) were analyzed. 3D FF in asymptomatic hips was lower than in symptomatic hips (Gmin: 17.8% vs. 19.8%; Gmed: 12.7% vs. 15.9% (all *p* ≤ 0.02)). Gmin had a higher 3D FF than Gmed (*p* < 0.001). Females had higher FF (asymptomatic and symptomatic Gmin: 19.4%, 21.8%; asymptomatic and symptomatic Gmed: 13.2%, 16.3%) than males (asymptomatic and symptomatic Gmin: 14.7%, 16.1%; asymptomatic and symptomatic Gmed: 11.8%, 14.9%) for both sides and muscles. Average WMV in asymptomatic hips for Gmin and Gmed were 77.2 cm^3^, 270.1 cm^3^ in females, and lower in males (both *p* < 0.001) with 107.1 cm^3^, 408.1 cm^3^, respectively.

**Conclusion:**

This study offers reference values for 3D FF and volumes of the Gmin and Gmed muscles in asymptomatic elderly hips, which are significantly lower than their GTPS counterparts, with succinctly higher fat fractions in females than males. Women showed significantly lower muscle volume for both muscles than men.

**Critical relevance statement:**

Volumetric fat fractions of gluteal muscles show significant symptoms and gender related differences, indicating their potential as an imaging biomarker in the common GTPS patient.

**Key Points:**

- In females, asymptomatic hips showed average volumetric fat fractions of 19% for Gmin and 13% for Gmed; with lower values in males, of 15% and 12%, respectively.
- Whole muscle volumes in asymptomatic hips for Gmin and Gmed were 77.2 cm^3^, 270.1 cm^3^ in females, and 107.1 cm^3^, 408.1 cm^3^ in males.
- Using volumetric fat fractions, abductor muscle fat content was significantly higher in symptomatic GTPS hips compared to asymptomatic hips.

**Graphical Abstract:**

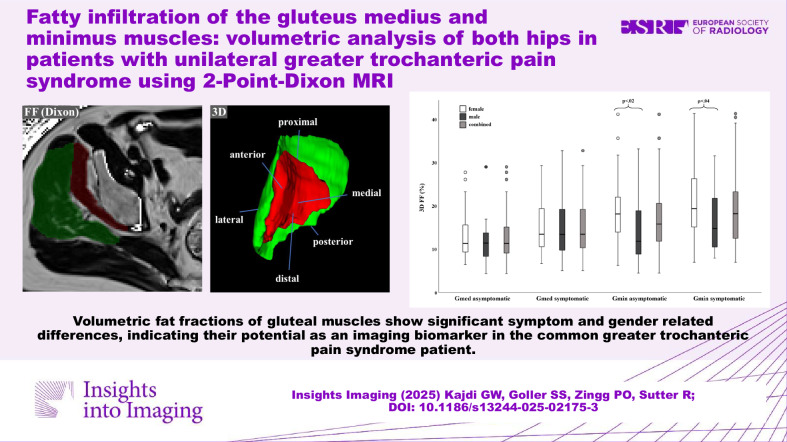

## Introduction

Greater trochanteric pain syndrome (GTPS) is both an acute and chronic, debilitating condition of the aging hip, affecting up to 25% of women above 50 years of age [1]. More than half of this demographic is affected unilaterally [2]. Risk factors include obesity, difference in leg length and prior hip arthroplasty [3, 4]. GTPS usually presents with thigh and/or buttock pain, pain to the touch and aggravation by exercise, lying on the side and sitting for a longer time [5]. Gmin and Gmed are the main hip abductors, often showing fatty infiltration in patients with hip osteoarthritis and patients with GTPS [6]. Trochanteric bursitis and tendon lesions of the Gmin and the Gmed are major causes contributing to the development of GTPS [5]. Surgery is reserved for refractory cases after failed conservative treatment, abductor tendon mass ruptures and failed revision. In these patients, a high extent of fatty infiltration of the muscles correlates with worse clinical outcome [7, 8]. With an increasing implementation of quantitative (e.g., fat fraction mapping) rather than semiquantitative (e.g., the ordinal Goutallier grades) image analysis, volumetric assessment of MRI datasets (e.g., muscle fat fractions and volume) is becoming an important part of radiomics, resulting in an imaging analysis that is more precise and has a higher reproducibility [9, 10]. To support clinical decision making, including objective diagnosis, monitoring of disease progression, and guidance for treatment planning (conservative vs. surgical management), the establishment of referential normal values for these anatomical structures in affected patient cohorts is required. Quantitative analysis of fatty infiltration using Dixon MRI has been extensively studied for the shoulder, allowing to define normal FF values and predict the risk of re-tears after arthroscopic rotator cuff repairs [11]. Few studies have evaluated the FF of the hip abductors using Dixon MRI, and this was mainly done in healthy adults below the age of 60 years [12, 13]. These normal FF and muscle volume values of the hip abductors are cohort-specific and do not represent the older age group where GTPS commonly occurs. Normal values for muscle volume and volumetric (3D) FF of the hip abductor muscles in an elderly population are not defined yet. Therefore, in this study, we evaluated the 3D FF, whole (WMV) and lean muscular volume (LMV) of the Gmin and Gmed muscles on both sides in a typical patient cohort with unilateral GTPS using 2-point Dixon MRI.

## Materials and methods

### Patient selection

Our patient cohort was part of two prior studies on different aspects of GTPS at our institution, for which ethics approval had been obtained (BASEC 2018-01354). The parent studies investigated degenerative hip abductor tendon lesions and cross-sectional FF without volumetric analysis [14, 15]. The current study focused on 3D muscle assessment and was carried out according to the principles of the Declaration of Helsinki and national ethical standards. All patients in the study had given their written informed consent, allowing their health-related data to be used for research.

**Fig. 1:**
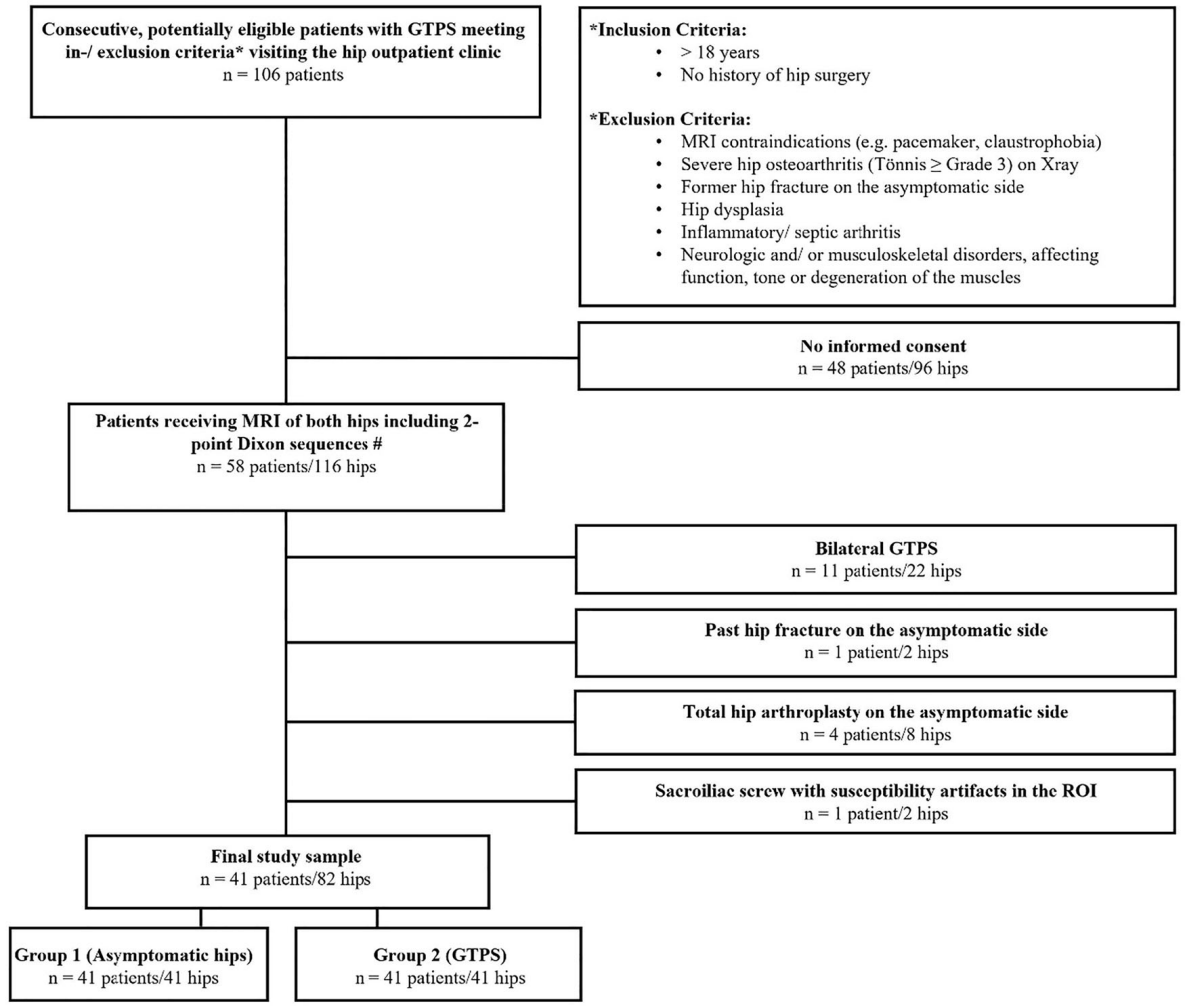
Flowchart illustrating the patient selection process. The final study sample consisted of 82 hips in 41 patients: Group 1 consisted of the asymptomatic 41 hips, whereas the contralateral symptomatic hips were put in Group 2. # MRI of both hips was acquired for a prior prospective study on greater trochanteric pain syndrome, as described in the manuscript text

The prospective parent study included 58 consecutive patients with GTPS from our orthopedic outpatient clinic. GTPS was clinically diagnosed based on characteristic lateral hip pain and focal tenderness over the greater trochanter by the orthopedic surgeon [14]. After exclusion of severe hip osteoarthritis on radiographs using the Tönnis score [16, 17], patients with conservatively treated refractory GTPS ≥ 6 months were clinically re-evaluated and received bilateral hip MRI at a follow-up of at least 36 months. Inclusion and exclusion criteria of the initial parent study are included in Fig. 1. Furthermore, 17 patients were excluded because of bilateral GTPS (*n* = 11), hip arthroplasty (*n* = 4), old hip fracture (*n* = 1), and because of strong susceptibility caused by a sacroiliac screw (*n* = 1), resulting in 41 included unilateral GTPS patients (Fig. 1).

### Magnetic resonance imaging

MRI was performed using a 3-T unit (Magnetom Prisma, Siemens Healthineers) (*n* = 41 patients/82 hips, 100%) with a dedicated 18-channel phased-array body coil following a standardized unilateral, non-contrast MRI protocol for each hip (Table 1).

**Table 1:** Sequence parameters for unilateral MRI of the hip abductor muscles at 3 T

	T2 TSE cor	T1 TSE sag	T1 TSE ax	2p-Dixon ax	T2 STIR ax
TR (ms)	4000	735	791	3.92	5000
TE (ms)	83	14	13	1.23	62
Bandwidth (Hz/px)	250	250	250	1000	290
Slices (*n*)	29	26	28	52	26
Slice thickness (mm)	4	4	6	5	7
Matrix	448 × 336	512 × 358	448 × 314	128 × 102	320 × 256
FOV (mm)	220	180	179	200	180
TA (min:s)	1:08	2:11	2:08	0:31	2:00

*cor* coronal, *Hz* Hertz, *FS* fat-saturated, *FOV* field of view, *sag* sagittal, *px* pixel, *TA* acquisition time, *TE* echo time, *TR* repetition time, *ax* axial, *TSE* turbo spin echo, *STIR* short tau inversion recovery

### Image analysis

#### Quantitative image analysis

**Fig. 2:**
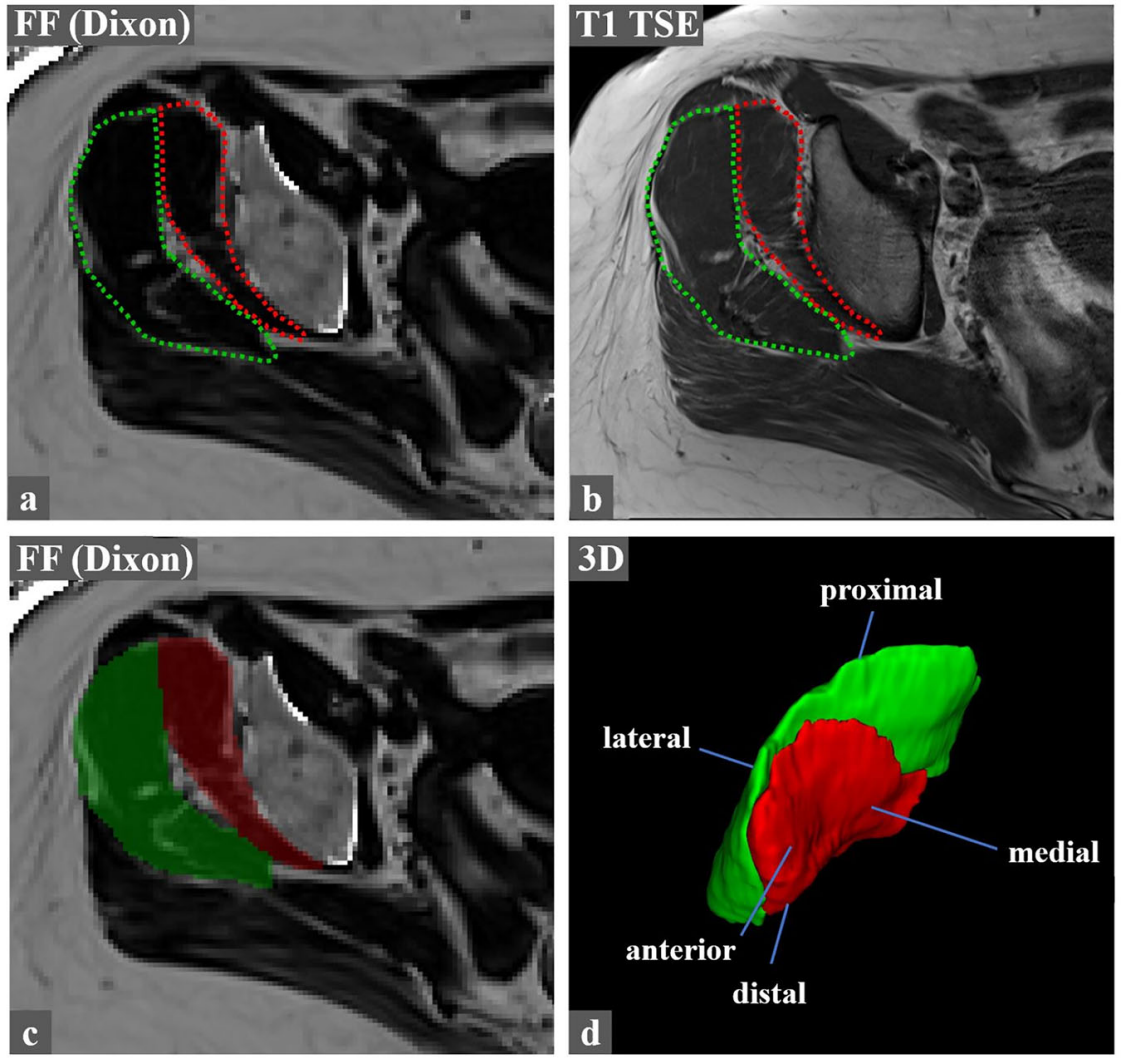
Illustration of the volumetric assessment of the abductor muscles in the right hip of a 58-year-old female patient with ipsilateral GTPS. Using ITK-Snap, the Gmin (red) and Gmed (green) were outlined on the Dixon-derived axial FF images (**a**). The corresponding axial T1 TSE sequence (**b**) aided in muscle delineation and in avoiding the inter-muscular fat plane carrying the deep branches of the superior gluteal artery. After the Gmin and Gmed were outlined separately on every axial slice, a semi-transparent volumetric color-overlay (Gmin: red; Gmed: green) on the axial Dixon-derived FF images was automatically superimposed (**c**) and translated into a separate 3D reconstruction (**d**) that contained the data of the mean 3D FF (in this case 12.9% for the Gmin and 12.6% for the Gmed muscle) and volume for each delineated muscle. FF, fat fractions; Gmin, gluteus minimus muscle; Gmed, gluteus medius muscle; GTPS, greater trochanteric pain syndrome; TSE, turbo spin echo

Volumetric image analysis was executed using ITK-Snap (version 4.2.0, Cognitica; available at https://www.itksnap.org) [18] and a picture archiving and communication system (PACS) workstation (Merlin, Phoenix-PACS). All studies were anonymized and independently evaluated by two musculoskeletal fellowship-trained radiologists (S.S.G., G.W.K., with 5 and 6 years of experience, respectively), with one reader repeating the analysis after 12 months for intra-reader assessment. Cases were reviewed in random order, and readers were blinded to clinical data. Image analysis was executed as follows: Using ITK-Snap [18], the Gmin and Gmed were manually segmented on all axial Dixon FF slices, supported by axial T1-weighted images for anatomical reference. Tendon insertions and the inter-muscular fat plane carrying the superior gluteal artery were excluded from the volumetric assessment, whereas fatty serrations at the muscle margins were included. After both the Gmin and Gmed muscles were outlined on all slices, automated visual 3D reconstructions were generated (Fig. 2). Based on the manually outlined volumetric dataset, the mean 3D FF and WMV for each muscle were then automatically computed by ITK-Snap. LMV was calculated, according to prior literature [12], as follows: LMV = WMV * (1 – 3D FF).

### Qualitative image analysis

In addition to volumetric analysis, peri-trochanteric MRI findings were retrospectively assessed by one reader (GWK). GTPS morphology was graded on an established ordinal scale (0 = no abnormality, 1 = trochanteric bursitis, 2 = gluteal tendinopathy, 3 = partial-thickness tear, 4 = full-thickness tear) according to Amin et al [19] and used as a semiquantitative severity score. This assessment was performed blinded on the same MRI datasets.

### Statistical analysis

Statistical analysis was performed in SPSS Statistics (v. 29, IBM). Normal vs. non-normal data distribution was assessed graphically (Q-Q plots) and with the Shapiro–Wilk test. Hips were categorized as asymptomatic or symptomatic, with gender-specific sub-analysis. Descriptive statistics included mean, median, standard deviation (SD), interquartile range (IQR), and range. Side-to-side differences were tested with the Wilcoxon signed-rank test, and gender-specific differences with the Mann–Whitney U test. Age and anthropometric measures were correlated with non-derived MRI parameters (3D FF, WMV) using Pearson correlation. Intra- and inter-reader agreement for 3D FF, WMV, and LMV was assessed using intraclass correlation coefficients (ICC) (two-way mixed-effects model, absolute agreement). Agreement was classified using established thresholds [20]. Associations between peri-trochanteric morphology and quantitative measures were explored using Spearman’s rank correlation. Morphology frequencies were reported descriptively. All statistical tests were two-sided, and a level of significance (α) of 0.05 was used.

## Results

### Demographic and patient characteristics

41 patients (mean age 65.0 ± 13.6 years, range 38–81, 27 females) with unilateral GTPS were included in the study. Patients had a mean BMI of 27.0 ± 4.7 kg/m^2^ (Table 2). GTPS was equally distributed between the left (*n* = 20 patients) and right (*n* = 21 patients) hip, without significant difference (*p* = 0.8). The Tönnis score for hip osteoarthritis was without significant differences between the asymptomatic and GTPS hip (*p* = 0.10), with a median Tönnis score of 1 bilaterally (range: 0–2 [left], 0–1 [right] respectively).

**Table 2:** Patient demographics of the study population

	All patients (*n* = 41)	Female patients (*n* = 27)	Male patients (*n* = 14)	*p*-value
Age (in years)*	65.0 (13.6)	68.3 (11.2)	58.2 (16.6)	**0.039**
Height (in cm)*	167.2 (9.5)	161.9 (6.2)	177.3 (5.9)	**< 0.001**
Weight (in kg)*	76.0 (17.1)	68.6 (13.0)	90.4 (14.9)	**< 0.001**
BMI (in kg/m^2^)*	27.0 (4.7)	26.2 (4.9)	28.7 (3.9)	0.083

Significant results (*p* < 0.05) are printed in bold

*BMI* body mass index

* Continuous data is given as mean (standard deviation)

### Quantitative analysis

#### Volumetric 2-point echo Dixon MRI-derived fat fractions (3D FF)

In females, the mean 3D FF was 19.4 ± 8.2% on the asymptomatic and 21.8 ± 9.4% on the GTPS side for the Gmin. Mean 3D FF for Gmed in females was 13.2 ± 5.7% on the asymptomatic and 16.3 ± 9.9% on the GTPS side. In males, the mean 3D FF was 14.7 ± 8.6% on the asymptomatic and 16.1 ± 6.6% on the GTPS side for the Gmin. Mean 3D FF for Gmed in males were 11.8 ± 6.3% on the asymptomatic and 14.9 ± 7.5% on the GTPS side (Supplementary Table 1).

**Fig. 3:**
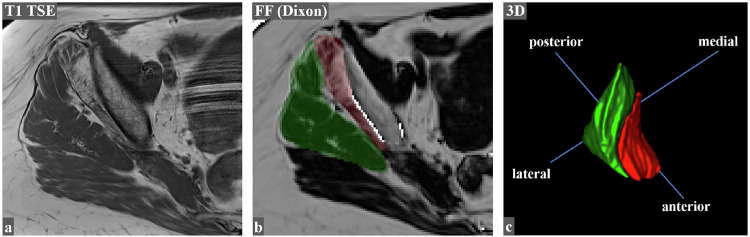
80-year-old female patient with unilateral GTPS on the right with a case of higher 3D FF of the Gmin compared to the Gmed muscle. On the T1w TSE (**a**), used for anatomic correlation, a much higher fatty infiltration of Gmin compared to the Gmed can be non-quantitatively observed. Muscle segmentation on the 2p-Dixon-derived FF-maps (**b**), revealed a 3D FF of 41.3% for the Gmin (red overlay) and of 14.8% for the Gmed (green overlay) muscle. The inter-muscular fat plane can be seen as a ridge on the cranial 3D perspective (**c**) between the Gmin (red) and the Gmed (green) muscle. FF, fat fractions; Gmin, gluteus minimus muscle; Gmed, gluteus medius muscle; GTPS, greater trochanteric pain syndrome; TSE, turbo spin echo. For educational purposes and better depiction of the underlying muscle anatomy on image **b**, with considerable fatty infiltration, the segmentation overlays were increased in transparency; artifacts along the cortical bone of the ilium were excluded from the segmentation

**Fig. 4:**
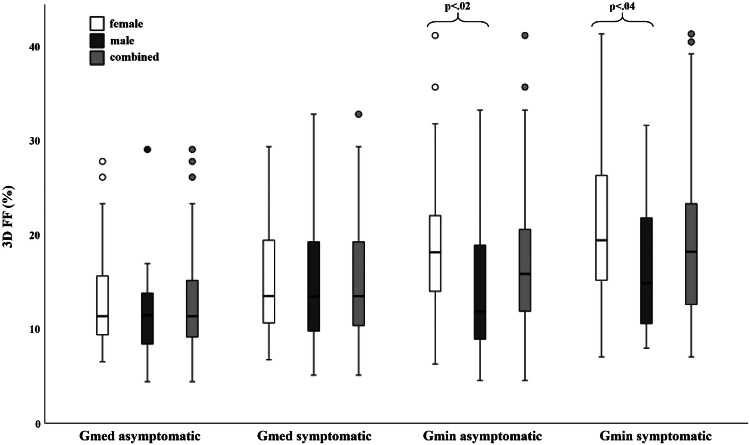
Volumetric fat fraction (3D FF) boxplots of the Gmin and Gmed muscle on the asymptomatic vs. symptomatic side in females (white), males (dark gray) and the overall study cohort with both genders combined (light gray). The minimum (lower whisker), first quartile (lower box margin), median (thick black line), third quartile (upper box margin), and maximum FF (upper whisker) of the Gmin and Gmed muscles are shown. Dots above the whiskers represent outliers. The brackets denote statistically significant differences between the genders with the corresponding *p*-value. Gmin, gluteus minimus muscle; Gmed, gluteus medius muscle

Mean 3D FF in asymptomatic hips of the overall study cohort were significantly lower than in symptomatic hips for both the Gmin (*p* = 0.02) and Gmed (*p* < 0.001) (Table 3). Gmin had significantly higher 3D FF than Gmed (*p* < 0.001), irrespective of gender and affected side (Fig. 3). Both the Gmin and Gmed showed higher 3D FF in females than in males, but this was only statistically significant for the Gmin (*p* ≤ 0.039), not for the Gmed (*p* ≥ 0.45) (Table 3, Fig. 4). A significant (*p* < 0.001), moderate to strong positive correlation between age and 3D FF (Gmin: r_s_ = 0.62; Gmed: r_s_ = 0.45) was detected for both muscles. A significant (*p* < 0.001), moderate correlation between BMI and 3D FF of the Gmed (r_s_ = 0.44) was found, while no such correlation was found for the Gmin (Supplementary Table 2).

**Table 3:** Side comparison of volumetric fat fractions (3D FF), whole (WMV) and lean muscle volume (LMV) for Gmin and Gmed

	Muscle		Asymptomatic hip	Symptomatic hip	*p*-value
3D FF* (%)	Gmin	Female	19.4 (8.2)	21.8 (9.4)	0.08
		Male	14.7 (8.6)	16.1 (6.6)	0.12
		Overall	17.8 (8.5)	19.8 (8.9)	**0.02**
	Gmed	Female	13.2 (5.7)	16.3 (9.9)	**< 0.001**
		Male	11.8 (6.3)	14.9 (7.5)	**0.03**
		Overall	12.7 (5.9)	15.9 (9.1)	**< 0.001**
WMV* (cm^3^)	Gmin	Female	77.16 (16.6)	74.1 (16.8)	0.05
		Male	107.1 (24.5)	107.5 (29.1)	0.81
		Combined	87.4 (24.1)	85.5 (26.7)	0.27
	Gmed	Female	270.1 (49.3)	278.9 (58.2)	0.11
		Male	408.0 (77.2)	405.4 (79.9)	0.78
		Combined	317.2 (88.9)	322.1 (89.3)	0.31
LMV* (cm^3^)	Gmin	Female	61.0 (16.8)	58.3 (16.4)	0.10
		Male	92.0 (25.1)	90.9 (26.5)	0.78
		Combined	71.6 (24.7)	69.5 (25.8)	0.27
	Gmed	Female	233.7 (41.1)	232.6 (51.8)	0.55
		Male	359.7 (69.8)	344.3 (70.7)	0.30
		Combined	276.7 (80.0)	270.7 (79.0)	0.73

Significant results (*p* < 0.05) are printed in bold

^*^ Data are given as mean (standard deviation)

### WMV in females, males and in side comparison

In females, the mean WMV was 77.2 ± 16.6 cm^3^ on the asymptomatic and 74.1 ± 16.8 cm^3^ on the GTPS side for the Gmin. Mean WMV for Gmed in females was 270.1 ± 49.3 cm^3^ on the asymptomatic and 278.9 ± 58.2 cm^3^ on the GTPS side. In males, the mean WMV was 107.1 ± 24.5 cm^3^ on the asymptomatic and 107.5 ± 29.1 cm^3^ on the GTPS side for the Gmin. Mean WMV for Gmed in males were 408.0 ± 77.2 cm^3^ on the asymptomatic and 405.4 ± 80.0 cm^3^ on the GTPS side (Supplementary Table 1).

**Fig. 5:**
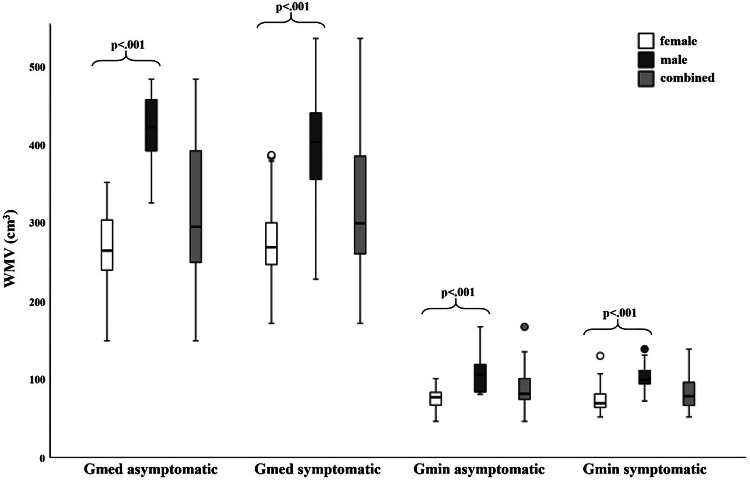
Whole muscle volume (WMV) boxplots of the Gmin and Gmed on the asymptomatic and symptomatic side in females (white), males (dark gray) and both genders combined (light gray). The minimum (lower whisker), first quartile (lower box margin), median (thick black line), third quartile (upper box margin), and maximum FF (upper whisker) of the Gmin and Gmed muscles are shown. Dots above the whiskers represent outliers. The brackets denote statistically significant differences between the genders with the corresponding *p*-value. Gmin, gluteus minimus muscle; Gmed, gluteus medius muscle

Mean WMV of the Gmin and Gmed did not differ significantly between the asymptomatic and GTPS hips, irrespective of gender (all *p* ≥ 0.05) (Table 3). Both Gmin and Gmed showed significantly lower WMV in females than in males (all *p* < 0.001), irrespective of hip side (Fig. 5).

### LMV in females, males and in side comparison

In females, the mean LMV was 61.0 ± 16.8 cm^3^ on the asymptomatic and 58.3 ± 16.4 cm^3^ on the GTPS side for the Gmin. Mean LMV for Gmed in females was 233.7 ± 41.1 cm^3^ on the asymptomatic and 232.6 ± 51.8 cm^3^ on the GTPS side. In males, the mean LMV was 92.0 ± 25.1 cm^3^ on the asymptomatic and 90.9 ± 26.5 cm^3^ on the GTPS side for the Gmin. Mean LMV for Gmed in males was 359.71 ± 69.8 cm^3^ on the asymptomatic and 344.3 ± 70.7 cm^3^ on the GTPS side (Supplementary Table 1).

**Fig. 6:**
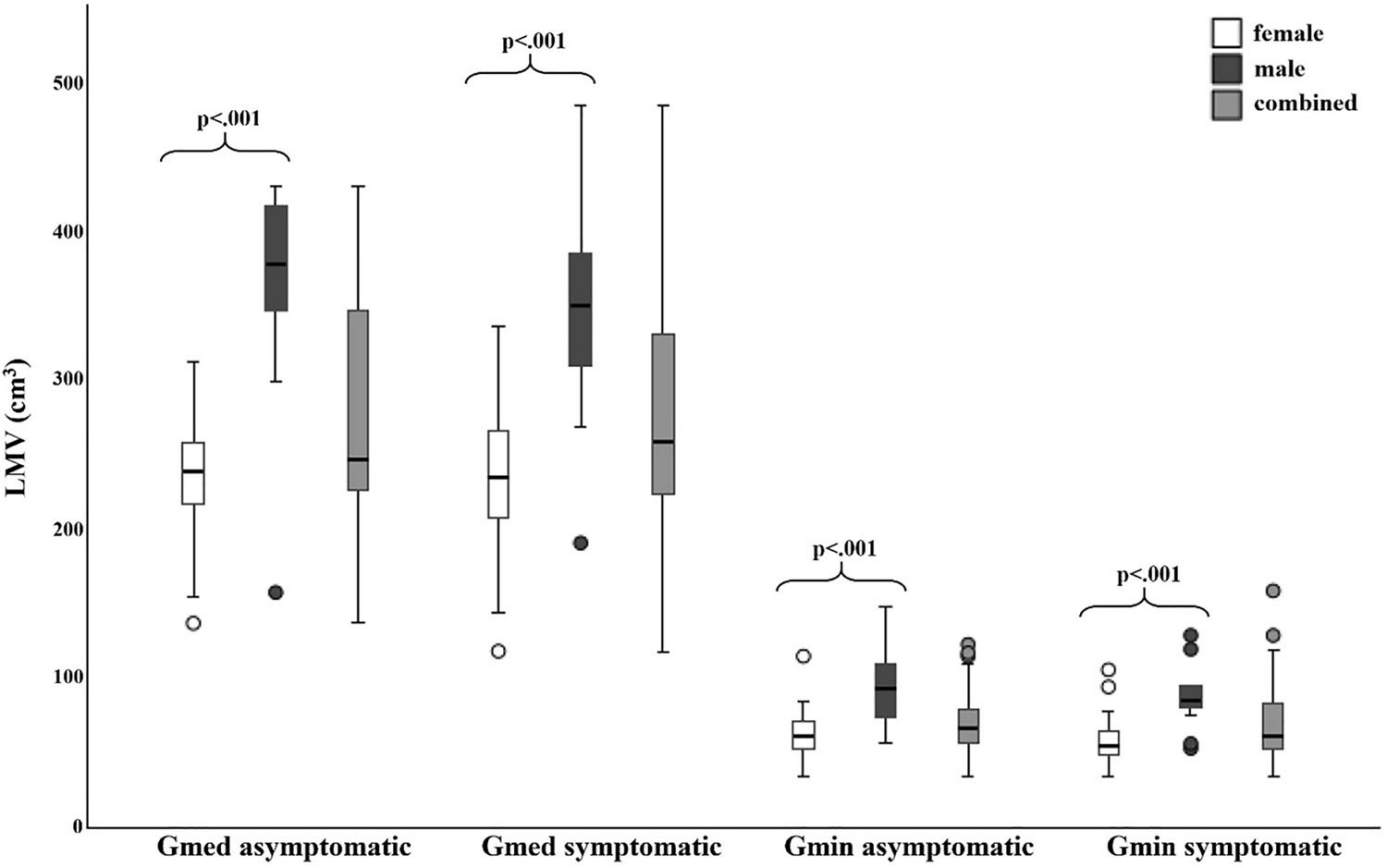
Lean muscle volume (LMV) boxplots of the Gmin and Gmed muscle on the asymptomatic and symptomatic side in females (white), males (dark gray) and the overall study cohort with both genders combined (light gray). The minimum (lower whisker), first quartile (lower box margin), median (thick black line), third quartile (upper box margin), and maximum FF (upper whisker) of the Gmin and Gmed muscles are shown. Dots above and below the whiskers represent outliers. The brackets denote statistically significant differences between the genders with the corresponding *p*-value. Gmin, gluteus minimus muscle; Gmed, gluteus medius muscle

Mean LMV of the Gmin and Gmed did not differ significantly between the asymptomatic and GTPS hips, irrespective of gender (all *p* ≥ 0.102) (Table 3). Both the Gmin and Gmed showed significantly lower LMV in females than in males (all *p* < 0.001), irrespective of the hip side (Fig. 6).

Intra-/inter-reader reliability was almost perfect for 3D FF, WMV, and LMV in both Gmin and Gmed (Table 4).

**Table 4:** Intra- and inter-reader agreement for volumetric fat fraction (3D FF), whole (WMV) and lean muscle volume (LMV) for Gmin and Gmed

Intra-reader agreement				
Gmin				
Parameter	Mean ± SD (1st measurement)	Mean ± SD (2nd measurement)	ICC (95% CI)	Interpretation
3D FF (%)	18.8 ± 8.7	18.9 ± 9.2	0.98 (0.96–0.98)	Almost perfect
WMV (cm^3^)	86.5 ± 25.3	86.0 ± 25.1	0.98 (0.97–0.99)	Almost perfect
LMV (cm^3^)	70.5 ± 25.1	70.4 ± 24.1	0.96 (0.94–0.98)	Almost perfect
Gmed				
3D FF (%)	14.3 ± 7.7	15.3 ± 9.1	0.94 (0.89–0.96)	Almost perfect
WMV (cm^3^)	319.6 ± 88.5	317.2 ± 96.8	0.95 (0.92–0.97)	Almost perfect
LMV (cm^3^)	273.7 ± 78.9	268.7 ± 87.8	0.95 (0.92–0.97)	Almost perfect
Inter-reader agreement				
Gmin				
**Parameter**	**Mean** **±** **SD (1st reader)**	**Mean** **±** **SD (2nd reader)**	**ICC (95% CI)**	**Interpretation**
3D FF (%)	18.8 ± 8.7	20.2 ± 10.2	0.91 (0.86–95)	Almost perfect
WMV (cm^3^)	86.5 ± 25.3	87.0 ± 25.9	0.96 (0.93–0.97)	Almost perfect
LMV (cm^3^)	70.5 ± 25.1	70.3 ± 25.3	0.94 (0.90–0.96)	Almost perfect
Gmed				
3D FF (%)	14.3 ± 7.7	16.6 ± 9.7	0.88 (0.70–0.94)	Almost perfect
WMV (cm^3^)	319.6 ± 88.5	317.5 ± 98.4	0.94 (0.91–0.96)	Almost perfect
LMV (cm^3^)	273.7 ± 78.9	273.7 ± 78.9	0.93 (0.89–0.96)	Almost perfect

*CI* confidence interval, *SD* standard deviation

### Qualitative analysis

Morphological changes were more common in symptomatic hips (Table 5), most frequently partial-thickness tendon tears (48.8%). Asymptomatic hips more often showed no abnormality (7.3%) or milder findings such as bursitis (12.2%), and most frequently tendinopathy (43.9%). The morphology severity score demonstrated a moderate, positive correlation with 3D FF for both Gmed (r_s_ = 0.49) and Gmin (r_s_ = 0.57; both *p* < 0.001), whereas no significant association was found with WMV or LMV for either muscle (all *p* ≥ 0.12).

**Table 5:** Peri-trochanteric morphology changes on MRI in symptomatic vs. asymptomatic hips

MRI morphology category	Symptomatic hips (*n*, %)	Asymptomatic hips (*n*, %)
No peri-trochanteric abnormality	0 (0%)	3 (7.3%)
Trochanteric bursitis	4 (9.8%)	5 (12.2%)
Gluteal tendinopathy	16 (39.0%)	18 (43.9%)
Partial gluteal tendon tear	20 (48.8%)	15 (36.6%)
Full-thickness gluteal tendon tear	1 (2.4%)	0 (0%)

## Discussion

In this study, normal values for volumetric fat fractions (3D FF), whole muscle volume (WMV) and lean muscle volume (LMV) of the abductor muscles of asymptomatic hips were elucidated in a representative cohort of patients with common unilateral greater trochanteric pain syndrome (GTPS). GTPS hips showed significantly higher 3D FF in both the Gmin and Gmed muscles compared to asymptomatic hips, while having comparable muscle volumes. Gender-specific differences were also elucidated.

The typical GTPS demographic was well represented by our cohort, with a comparable mean age in the literature (62.8 years vs. 65.1 years in our cohort) and a known female predilection [21, 22]. Mean height, weight and BMI of our cohort were close to the age- and gender-specific 50th percentile reported for Caucasians [23].

Mean 3D FF in asymptomatic hips of the overall study cohort was significantly lower than in symptomatic hips for both the Gmin (*p* = 0.02) and Gmed (*p* < 0.001), an observation that was obscured in prior studies with assessment of fatty infiltration using the ordinal Goutallier classification [24]. Despite their common function as the main hip abductors, the significant difference in 3D FF between the Gmin and Gmed clearly advocates for their individual quantitative assessment. This muscle-specific vulnerability was also reflected in anthropometric correlations: BMI showed a moderate, significant association with Gmed 3D FF, however, not with Gmin 3D FF. This suggests that the Gmed is more susceptible to BMI-related mechanical load, while Gmin is more likely susceptible to age- or degeneration-related factors. Surgical GTPS study cohorts further support this pattern, as Gmed tears frequently occur in isolation [25, 26], whereas Gmin tears typically accompany Gmed tears rather than appearing alone [26].

The 3D FF for both the Gmin and Gmed were higher in females than in males, irrespective of the side. However, these gender-specific differences only reached significance for the Gmin muscle (*p* < 0.003) and not for the Gmed muscle (*p* = 0.39). This is most likely due to the on average bigger difference in 3D FF between females and males for the Gmin muscle of 5–6%, compared to 1–2% for the Gmed muscle. The difference in FF of the hip abductors between genders does not universally apply to other patient cohorts: A study on middle-aged, healthy cyclists (*n* = 87) challenged the preexisting notion of gender-based differences in intramuscular fat content and muscle mass of the gluteus muscles [13]: Belzunce et al showed gender equivalence in intramuscle fat content of the gluteal muscles when matched for BMI, but significantly higher muscle volumes in men compared to women. Similarly, in our study, men showed higher muscle volumes for both the Gmin and Gmed compared to women (*p* < 0.001). Yet, despite no significant differences in BMI between the genders (*p* = 0.083), we found a significant difference in 3D FF for the Gmin between females and males, contrasting Belzunce et al’s results [13]. This may reflect sex-specific biomechanical characteristics, such as greater trochanteric offset [27], pelvic width [28], and hormone-associated post-menopausal tendon and muscle changes [29, 30], known to predispose women to gluteal tendon degeneration and fatty muscle infiltration [31].

From a biomarker perspective, the clinical utility of quantitative MRI depends not on statistical significance alone but on interpretable change metrics. Although 3D FF was significantly higher in symptomatic hips, inter-individual variability and overlap preclude a robust universal threshold at this stage. Our data instead support a patient-specific interpretation, using the contralateral asymptomatic hip as an internal reference, where normalization toward this individual baseline may represent a more meaningful therapeutic and longitudinal monitoring target than exceeding a fixed cut-off.

Mean 3D FF for Gmin and Gmed in asymptomatic hips of our overall study cohort aligned well and were 2% and 7% higher, respectively, than the mean FF reference values previously reported for younger, healthy adults [12]. This implies that fatty infiltration of the hip abductor muscles increases with age even in asymptomatic hips, further supported by the strong positive correlation between age and 3D FF among our study cohort in both muscles.

In contrast to the 3D FF, WMV did not differ between the asymptomatic and GTPS side in either gender, consistent with Retchford et al, who also found no muscle volume differences in patients with hip pain versus controls [24]. The WMV of the Gmin was comparable to the WMV reported for young, healthy adults in a prior study, whereas the mean WMV of Gmed in our study was 6.7% lower than the pre-reported WMV in young adults [12]. Both findings reinforce the assumption that fatty infiltration and sarcopenia are independently associated pathophysiologic processes [32], that each affects the Gmin and Gmed to different extents, and hence should be assessed separately. The significantly higher WMV for both the Gmin and Gmed in males compared to females aligns with the literature [12].

LMV did not differ between symptomatic and asymptomatic hips and therefore did not add diagnostic value beyond 3D FF and WMV. Its comparable behavior to WMV nevertheless helped confirm that no systematic overestimation of 3D FF occurred during manual segmentation.

The almost perfect reproducibility for 3D FF, WMV and LMV of the gluteal muscles (ICC 0.89–0.98) aligns with prior reports for the rotator cuff [33] and lumbar multifidus muscles [34], underscoring the robustness of Dixon-based volumetric biomarkers across anatomic regions, as a key prerequisite for an imaging biomarker. Beyond diagnosis, quantitative assessment of gluteal muscle quality may support structured GTPS care: early fatty change may guide targeted hip-abductor strengthening, whereas more advanced fatty degeneration could signal limited reversibility and favor earlier surgical referral. Similar Dixon-based FF metrics already inform treatment decisions in rotator cuff [11] and paraspinal muscle disease [35].

Beyond quantitative muscle analysis, peri-trochanteric morphology was also considered. Symptomatic hips more often demonstrated tendinopathy or partial tears, and the moderate association between morphology and intramuscular fat supports that advancing gluteal tendon pathology accompanies greater fatty degeneration, whereas muscle volume showed no such relationship.

Limitations of this study need to be addressed. Our cohort only encompassed 41 patients. However, this is the first study with a combined report of volumetric FF and muscle volumes of asymptomatic hip abductor muscles compared to symptomatic hips in an age cohort typical for GTPS. All patients suffered from unilateral GTPS: Matched hips were asymptomatic, but did not perfectly represent healthy hips. Manual volumetric segmentation remains time-intensive and not yet suitable for routine use. With limited sensitivity of visual scales in GTPS (e.g., the ordinal Goutallier scale) [15], cross-sectional FF measurements [36, 37] may offer a more time-efficient interim alternative with strong correlation to the respective 3D FF until automated tools become available. All patients had chronic GTPS (≥ 6 months), but exact symptom duration, activity level, and movement restrictions were not systematically recorded. Thus, the relative contribution of disuse versus chronic degenerative change to fatty atrophy remains unclear. Possible confounders, significantly correlating with 3D FF and WMV, like higher height, weight and age in males, were present. However, these differences represent the clinical reality of the patient demographic [21–23], and do not diminish the external validity of our data.

## Conclusion

This study offers reference values for fat fractions and volumes of the Gmin and Gmed muscles in asymptomatic hips in typical GTPS patients, which are significantly lower than their symptomatic counterparts, with succinctly higher fat fractions in females compared to males. Females showed significantly lower muscle volume for both muscles.

## Supplementary information

ELECTRONIC SUPPLEMENTARY MATERIAL

## Data Availability

The data analyzed during this study are available from the corresponding author upon reasonable request.

## Abbreviations

3D FF: Volumetric fat fraction
BMI: Body mass index
FF: Fat fraction
Gmed: Gluteus medius (muscle)
Gmin: Gluteus minimus (muscle)
GTPS: Greater trochanteric pain syndrome
ICC: Intraclass correlation coefficient
LMV: Lean muscle volume
SD: Standard deviation
WMV: Whole muscle volume

## References

[1] Williams BS, Cohen SP (2009) Greater trochanteric pain syndrome: a review of anatomy, diagnosis and treatment. Anesth Analg 108:1662–1670 19372352 10.1213/ane.0b013e31819d6562

[2] Segal NA, Felson DT, Torner JC et al (2007) Greater trochanteric pain syndrome: epidemiology and associated factors. Arch Phys Med Rehabil 88:988–992 17678660 10.1016/j.apmr.2007.04.014 PMC2907104

[3] Nissen MJ, Genevay S (2015) [Greater trochanteric pain syndrome]. Rev Med Suisse 11:585–590 25946869

[4] Plinsinga ML, Ross MH, Coombes BK, Vicenzino B (2019) Physical findings differ between individuals with greater trochanteric pain syndrome and healthy controls: a systematic review with meta-analysis. Musculoskelet Sci Pract 43:83–90 31369906 10.1016/j.msksp.2019.07.009

[5] Redmond JM, Chen AW, Domb BG (2016) Greater trochanteric pain syndrome. J Am Acad Orthop Surg 24:231–240 26990713 10.5435/JAAOS-D-14-00406

[6] Kivle K, Lindland ES, Mjaaland KE, Svenningsen S, Nordsletten L (2021) Gluteal atrophy and fatty infiltration in end-stage osteoarthritis of the hip: a case-control study. Bone Jt Open 2:40–47 33537675 10.1302/2633-1462.21.BJO-2020-0179.R1 PMC7842157

[7] Bogunovic L, Lee SX, Haro MS et al (2015) Application of the Goutallier/Fuchs rotator cuff classification to the evaluation of hip abductor tendon tears and the clinical correlation with outcome after repair. Arthroscopy 31:2145–2151 26188781 10.1016/j.arthro.2015.04.101

[8] Looney AM, Bodendorfer BM, Donaldson ST, Browning RB, Chahla JA, Nho SJ (2022) Influence of fatty infiltration on hip abductor repair outcomes: a systematic review and meta-analysis. Am J Sports Med 50:2568–2580 34495797 10.1177/03635465211027911

[9] Belzunce MA, Henckel J, Fotiadou A, Di Laura A, Hart A (2020) Automated measurement of fat infiltration in the hip abductors from Dixon magnetic resonance imaging. Magn Reson Imaging 72:61–70 32615150 10.1016/j.mri.2020.06.019

[10] Fischer M, Kustner T, Pappa S et al (2023) Identification of radiomic biomarkers in a set of four skeletal muscle groups on Dixon MRI of the NAKO MR study. BMC Med Imaging 23:104 37553619 10.1186/s12880-023-01056-9 PMC10408104

[11] Feuerriegel GC, Marcus RP, Sommer S, Wieser K, Bouaicha S, Sutter R (2024) Fat fractions of the rotator cuff muscles acquired with 2-point Dixon MRI: predicting outcome after arthroscopic rotator cuff repair. Invest Radiol 59:328–336 37707864 10.1097/RLI.0000000000001024 PMC11882186

[12] Belzunce MA, Henckel J, Di Laura A, Hart AJ (2022) Reference values for volume, fat content and shape of the hip abductor muscles in healthy individuals from Dixon MRI. NMR Biomed 35:e4636 34704291 10.1002/nbm.4636

[13] Belzunce MA, Henckel J, Di Laura A, Horga LM, Hart AJ (2023) Gender similarities and differences in skeletal muscle and body composition: an MRI study of recreational cyclists. BMJ Open Sport Exerc Med 9:e001672 37637483 10.1136/bmjsem-2023-001672 PMC10450064

[14] Schenk P, Dimitriou D, Rahm S et al (2023) Natural history of degenerative hip abductor tendon lesions. Am J Sports Med 51:160–168 36412545 10.1177/03635465221135759 PMC9810830

[15] Kajdi GW, Goller SS, Zingg PO, Sutter R (2025) Cross-sectional fat fraction analysis of the gluteus medius and minimus muscle in asymptomatic vs. symptomatic hips using 2-point Dixon MRI. BMC Musculoskelet Disord 26:764 40775346 10.1186/s12891-025-09043-7 PMC12333093

[16] Tonnis D, Heinecke A (1999) Acetabular and femoral anteversion: relationship with osteoarthritis of the hip. J Bone Joint Surg Am 81:1747–1770 10608388 10.2106/00004623-199912000-00014

[17] Kovalenko B, Bremjit P, Fernando N (2018) Classifications in brief: Tonnis classification of hip osteoarthritis. Clin Orthop Relat Res 476:1680–1684 30020152 10.1097/01.blo.0000534679.75870.5f PMC6259761

[18] Yushkevich PA, Piven J, Hazlett HC et al (2006) User-guided 3D active contour segmentation of anatomical structures: significantly improved efficiency and reliability. Neuroimage 31:1116–1128 16545965 10.1016/j.neuroimage.2006.01.015

[19] Amin WM, Abdelkerim AA (2022) Greater trochanteric pain syndrome: a simplified MRI approach. Egypt J Radiol Nucl Med 53:82

[20] Landis JR, Koch GG (1977) The measurement of observer agreement for categorical data. Biometrics 33:159–174 843571

[21] Bicket L, Cooke J, Knott I, Fearon A (2021) The natural history of greater trochanteric pain syndrome: an 11-year follow-up study. BMC Musculoskelet Disord 22:1048 34930192 10.1186/s12891-021-04935-w PMC8691027

[22] Fearon AM, Scarvell JM, Neeman T, Cook JL, Cormick W, Smith PN (2013) Greater trochanteric pain syndrome: defining the clinical syndrome. Br J Sports Med 47:649–653 22983121 10.1136/bjsports-2012-091565

[23] Fryar CD, Carroll MD, Gu Q, Afful J, Ogden CL (2021) Anthropometric reference data for children and adults: United States, 2015–2018. Vital Health Stat 3:1–44 33541517

[24] Retchford TH, Tucker KJ, Hart HF et al (2022) No difference in hip muscle volumes and fatty infiltration in those with hip-related pain compared to controls. Int J Sports Phys Ther 17:851–862 35949368 10.26603/001c.36528 PMC9340835

[25] Yee C, Wong M, Cohen D et al (2023) Labral tears and chondral lesions are common comorbidities identified during endoscopic repair of gluteal tendon tears for greater trochanteric pain syndrome: a systematic review. Arthroscopy 39:856–864.e851 35817376 10.1016/j.arthro.2022.06.031

[26] Longstaffe R, Dickerson P, Thigpen CA et al (2021) Both open and endoscopic gluteal tendon repairs lead to functional improvement with similar failure rates: a systematic review. J ISAKOS 6:28–34 33833043 10.1136/jisakos-2020-000474

[27] Viradia NK, Berger AA, Dahners LE (2011) Relationship between width of greater trochanters and width of iliac wings in tronchanteric bursitis. Am J Orthop 40:E159–E162 22022680

[28] Fearon A, Stephens S, Cook J et al (2012) The relationship of femoral neck shaft angle and adiposity to greater trochanteric pain syndrome in women. A case control morphology and anthropometric study. Br J Sports Med 46:888–892 22547561 10.1136/bjsports-2011-090744 PMC3597182

[29] Hansen M, Kjaer M (2016) Sex hormones and tendon. Adv Exp Med Biol 920:139–149 27535256 10.1007/978-3-319-33943-6_13

[30] Frizziero A, Vittadini F, Gasparre G, Masiero S (2014) Impact of oestrogen deficiency and aging on tendon: concise review. Muscles Ligaments Tendons J 4:324–328 25489550 PMC4241423

[31] Grimaldi A, Fearon A (2015) Gluteal tendinopathy: integrating pathomechanics and clinical features in its management. J Orthop Sports Phys Ther 45:910–922 26381486 10.2519/jospt.2015.5829

[32] Barry JJ, Lansdown DA, Cheung S, Feeley BT, Ma CB (2013) The relationship between tear severity, fatty infiltration, and muscle atrophy in the supraspinatus. J Shoulder Elbow Surg 22:18–25 22541866 10.1016/j.jse.2011.12.014

[33] Khanna R, Saltzman MD, Elliott JM et al (2019) Development of 3D method to assess intramuscular spatial distribution of fat infiltration in patients with rotator cuff tear: reliability and concurrent validity. BMC Musculoskelet Disord 20:295 31221138 10.1186/s12891-019-2631-z PMC6587235

[34] Rummens S, Bosch S, Dierckx S et al (2022) Reliability and agreement of lumbar multifidus volume and fat fraction quantification using magnetic resonance imaging. Musculoskelet Sci Pract 59:102532 35245881 10.1016/j.msksp.2022.102532

[35] Wang S, Wang Y, Wang Z et al (2025) Comparative analysis of the correlation between paraspinal muscles fat infiltration and vertebral bone quality in patients with lumbar degenerative diseases. Eur Spine J 34:4486–4495 40659966 10.1007/s00586-025-09142-y

[36] Roach KE, Bird AL, Pedoia V, Majumdar S, Souza RB (2024) Automated evaluation of hip abductor muscle quality and size in hip osteoarthritis: localized muscle regions are strongly associated with overall muscle quality. Magn Reson Imaging 111:237–245 38636675 10.1016/j.mri.2024.04.025

[37] Perraton Z, Lawrenson P, Mosler AB et al (2022) Towards defining muscular regions of interest from axial magnetic resonance imaging with anatomical cross-reference: a scoping review of lateral hip musculature. BMC Musculoskelet Disord 23:533 35658932 10.1186/s12891-022-05439-x PMC9166386

