# Supplementary material for: Fatty infiltration of the gluteus medius and minimus muscles: volumetric analysis of both hips in patients with unilateral greater trochanteric pain syndrome using 2-point-Dixon MRI
Source: Insights Imaging. 2025 Dec 22;16:282. doi: 10.1186/s13244-025-02175-3 (PMC12722596; doi:10.1186/s13244-025-02175-3)
Supplement: Supplementary file 1 — ELECTRONIC SUPPLEMENTARY MATERIAL [file 13244_2025_2175_MOESM1_ESM.pdf]

Fatty infiltration of the gluteus medius and minimus muscles: Volumetric analysis of both hips in patients with unilateral greater trochanteric pain syndrome using 2-Point-Dixon MRI

ELECTRONIC SUPPLEMENTARY MATERIAL

Supplementary Table 1: Mean and median values for volumetric fat fraction (3D FF), whole (WMV) and lean (LMV) muscle volume for Gmin and Gmed.

		3D FF (%)		WMV (cm^3)		LMV (cm^3)	
		Mean (SD)	Median (IQR)	Mean (SD)	Median (IQR)	Mean (SD)	Median (IQR)
Asymptomatic							
Gmin	female	19.4(8.2)	18.1(8.4)	77.2(16.6)	77.1(17.2)	61.0(16.8)	60.9(20.7)
	male	14.7(8.6)	11.9(10.4)	107.1(24.5)	105.8(37.6)	92.0(25.1)	92.1(37.6)
	overall	17.8(8.5)	15.9(9.4)	87.4(24.1)	81.4(31.0)	71.6(24.7)	65.5(25.4)
Gmed	female	13.2(5.7)	11.4(6.9)	270.1(49.3)	264.7(78.0)	233.7(41.1)	236.6(58.9)
	male	11.8(6.3)	11.5(6.7)	408.0(77.2)	422.7(72.3)	359.7(69.8)	373.2(72.5)
	overall	12.7(5.9)	11.4(6.6)	317.2(88.9)	295(146.7.3)	276.7(79.6)	243.9(122.8)
Symptomatic							
Gmin	female	21.8(9.4)	19.4(13.6)	74.1(16.8)	69.4(20.5)	58.3(16.4)	54.6(18.3)
	male	16.1(6.6)	14.9(11.5)	107.5(29.1)	99.7(22.3)	90.9(26.5)	84.5(21.8)
	overall	19.8(8.9)	18.1(11.1)	85.5(26.7)	78.3(30.2)	69.5 (25.8)	60.7(30.0)
Gmed	female	16.3(9.9)	13.5(9.1)	278.9(58.2)	269.2(60.5)	232.6(51.8)	232.0(64.3)
	male	14.9(7.5)	13.5(10.2)	405.4(79.9)	403.8(92.1)	344.3(70.7)	345.6(84.3)
	overall	15.9(9.1)	13.5(9.1)	322.1(89.3)	299.7(127.3)	270.7(79.0)	255.5(106.4)

Gmin = gluteus minimus muscle ; Gmed = gluteus medius muscle ; SD = standard deviation; IQR = interquartile range

Supplementary Table 2: Correlation of age, height, weight and BMI with volumetric fat fraction (3D FF) and whole muscle volume (WMV) for Gmin and Gmed respectively.

**Gmin**		**Spearmans ρ (r_s)[1]**	**P-value[2]**
3D FF	Age	.62	**<.001**
	Height	-.31	**.005**
	Weight	-.13	.24
	BMI	.03	.80
WMV	Age	-.43	**<.001**
	Height	.80	**<.001**
	Weight	.57	**<.001**
	BMI	.26	**.02**
**Gmed**		**Spearmans ρ (r_s)[1]**	**P-value[2]**
3D FF	Age	0.45	**<.001**
	Height	-.16	.16
	Weight	.25	**.023**
	BMI	.44	**<.001**
WMV	Age	-.16	.15
	Height	.65	**<.001**
	Weight	.76	**<.001**
	BMI	.54	**<.001**

[1]Correlation is categorized as follows: .00-.19 = very weak; .20-.39 = weak; .40-.59 = moderate; .60-.79 = strong; .80-1.0 = very strong; where a positive sign denotes a direct and a negative sign denotes an inverse correlation. [2]Significant results (P<.05) are bolded

BMI = body mass index; Gmin = gluteus minimus muscle; Gmed = gluteus medius muscle
